# Analysis of Blame, Guilt, and Shame Related to Body and Body Weight and Their Relationship with the Context of Psychological Functioning Among the Pediatric Population with Overweight and Obesity: A Systematic Review

**DOI:** 10.3390/nu17111763

**Published:** 2025-05-23

**Authors:** Kamila Czepczor-Bernat, Marcela Mikulska, Paweł Matusik

**Affiliations:** Department of Pediatrics, Pediatric Obesity and Metabolic Bone Diseases, Faculty of Medical Sciences in Katowice, Medical University of Silesia, 40-055 Katowice, Poland; pmatusik73@gmail.com

**Keywords:** blame, guilt, shame, psychological functioning, obesity, overweight, childhood

## Abstract

**Background/Objectives:** There is scientific evidence showing that body- and/or body weight-related blaming, guilting, and shaming continue to be both promoted and tolerated in many societal contexts, including schools and healthcare settings. A deeply ingrained belief still prevails among many individuals that inducing these negative emotions can serve as a motivator for children and adolescents to engage in obesity treatment. Therefore, the aim of this systematic review is to examine these emotions (blame, guilt, shame) in relation to body weight and their impact on psychological functioning within the pediatric population affected by overweight and obesity. **Methods:** Articles were searched up using PubMed and Web of Science in June 2023 and March 2025. The search was conducted without limiting the years of publication. The inclusion criteria included the following: (1) pediatric samples, (2) full text available, and (3) original research articles. Articles were excluded if they were editorials, letters, replies from authors, review articles, and articles without a full text. **Results:** The initial search returned 199 results. A total of 16 articles were included in the study. Analysis of the collected records revealed associations between body- or weight-related blame, guilt, and shame and various aspects of psychological functioning in the pediatric population such as (a) interpersonal context (e.g., social stigma, bullying, teasing history, social connectedness, weight-related language used by parents in conversations with children and adolescents; (b) intrapsychic context—relationship with eating and food (e.g., binge eating, dietary restraint, emotional eating, and the risk of developing eating disorders); (c) intrapsychic context—self-perception (e.g., self-esteem, feelings of worthlessness, self-compassion, self-efficacy, perceived control); (d) intrapsychic context—emotional functioning (e.g., emotional distress, anxiety, depression, emotion regulation strategies); and (e) intrapsychic context—additional psychological factors (e.g., mindfulness, quality of life, willingness to seek help, and motivation for both help-seeking and sustaining successful lifestyle changes). **Conclusions:** Understanding the dynamics of body- and/or weight-related blame, guilt, and shame among children and adolescents with overweight and obesity is essential for developing effective support systems that enhance their well-being and psychological resilience in both the prevention and treatment of obesity. Further research is needed to explore the relationships between body- and weight-related blame, guilt, and shame and psychological functioning in pediatric populations with overweight and obesity, including the dynamics of child–parent–healthcare provider interactions, the context of parenting skills and attitudes that support the child during obesity treatment, the long-term consequences of body- and weight-related blame, guilt, and shame, the relationship between healthcare providers’ tendencies to engage in body- and weight-related shaming or blaming and their communication skills and mental well-being (e.g., levels of professional burnout, emotion regulation skills, and personality traits), as well as the influence of social media on body- and weight-related shame, guilt, and blame.

## 1. Introduction

The development of overweight and obesity during childhood and adolescence significantly increases the risk of numerous challenges and comorbidities, including those within the psychosocial domain [1,2,3,4]. Extensive research to date suggests that a key contributor to impairments in social functioning and mental health is the social stigma associated with body weight, along with the resulting daily exposure to discrimination and mistreatment [5,6]. Alberga et al. define weight bias as “(…) negative weight-related attitudes, beliefs, assumptions, and judgments toward individuals who are overweight and obese (…)” [7]. Puhl and Heuer further emphasize that this bias manifests through ridicule, exclusion, shaming, and patronization of individuals with overweight or obesity [8]. Among children and adolescents, such experiences can lead to persistent feelings of blame, guilt, and shame related to their body and weight [9,10,11]. It is crucial to examine the relationship between body weight-related blame, guilt, and shame and psychological well-being, as the presence and heightened intensity of these negative emotions significantly increase the risk of various mental health disorders, including depression, anxiety disorders, and eating disorders [1,3,4,12,13,14]. For instance, Puhl and Lessard report that 25–50% of adolescents experience body weight-related discrimination [15], while research by Cerolini et al. indicates that 37% of adolescents have encountered body shaming [12]. Compared to their peers who have not undergone such experiences, these individuals report higher levels of symptom severity and greater internalization of weight bias.

Moreover, responsibility for managing body weight is often disproportionately placed on the child, positioning them as the primary—or even sole—“culprit” in the development of obesity. This attribution can contribute to the internalization of weight stigma, fostering persistent feelings of blame, shame, and guilt [16,17,18]. Such emotions not only undermine motivation to initiate obesity treatment but also serve as significant barriers to adopting healthier lifestyle habits [19]. Their chronic persistence is further associated with poorer overall mental well-being and a depletion of the emotional resources necessary for sustained engagement in the treatment process—blame, guilt, and shame functioning as a “resource thief” [10]. Analyzing this issue is particularly important given that body-related blaming, guilting, and shaming continue to be both promoted and tolerated in many societal contexts, including schools and healthcare settings [20,21]. A deeply ingrained belief still prevails among many individuals that inducing these negative emotions can serve as a motivator for children and adolescents to engage in obesity treatment [13,22,23]. Evidence supporting this concern can be found, for instance, in the findings of Phelan et al., which indicate that weight bias among healthcare professionals diminishes the effectiveness of obesity treatment [24].

Given the growing prevalence of obesity among children and adolescents—rising from approximately 2% (31 million individuals) in 1990 to around 8% (160 million individuals) in 2022 [25]—there is an ongoing search for strategies to enhance the effectiveness of prevention and treatment efforts. Increasing attention is being directed toward social and psychological factors, as research indicates that a sense of self-efficacy and an internal locus of control can facilitate positive changes in health behaviors, whereas stigmatization, (self-)blame, body shaming, and guilt tend to have the opposite effect [12,19,26,27,28,29]. As previously discussed in the context of the “resource thief” concept, internalized weight stigma –rooted in body- and weight-related stereotypes—negatively impacts body image and psychological well-being. This growing culture of body blaming, shaming, and guilting exacts a substantial toll on mental health, increasing the likelihood of depression, low self-esteem, distorted body image, and maladaptive beliefs about the ability to change health behaviors [10,12,19,26,27,28,29]. These maladaptive beliefs often manifest through cognitive distortions such as all-or-nothing thinking, overgeneralization, and catastrophizing. Furthermore, they contribute to the development of binge–restrict cycles, in which individuals engage in extreme, unhealthy weight-control practices—such as fasting and very low-calorie diets—that heighten the risk of eating disorders [12,30,31,32]. Such psychological and behavioral patterns significantly hinder the effectiveness of obesity treatment. Notably, longitudinal studies further suggest that negative emotional experiences related to weight can predict the development of overweight and obesity in adulthood, as well as the emergence of various eating disorder symptoms [33,34]. It should be emphasized here that while obesity is not classified as a mental disorder, it may of be related to behaviors and psychological factors common to eating disorders. In particular, in patients with obesity we can observe [11,12,14,30,31,32] the following: (a) episodes of eating large quantities of food, often quickly and to the point of discomfort, accompanied by feelings of loss of control and shame (which may be associated with binge-eating disorder), (b) consuming food in response to emotion rather than physical hunger (which may be associated with emotional eating), (c) binge–restrict cycles (which may be associated with, among others, bulimia nervosa). Obesity and eating disorders may co-occur and share a wide range of common risk factors regarding psychosocial functioning and lifestyle factors (e.g., low self-esteem, body dissatisfaction, depression, anxiety, trauma/abuse history, sedentary lifestyle and limited access to healthy foods and recreational spaces, poor sleep habits, modeling of disordered eating in the context of family dynamics, unrealistic body image ideals, and weight stigma in the media) [1,4,11,12,14,30,31,32]. Preventing these harmful effects is particularly critical during childhood and adolescence—a developmental period in which personality, identity, self-esteem, and a sense of self-efficacy are being shaped, and when mechanisms such as social comparison and the need for social approval play a pivotal role in psychological development [2,35]. Preventive measures should include, among others, individual-level prevention (e.g., promoting a healthy relationship with food, body, and physical activity), school and family-level (e.g., education and parental modeling of healthy behaviors, anti-bullying activities), community and policy-level prevention (e.g., improving access to healthy foods and safe spaces for physical activity, limiting marketing of unhealthy foods to children, and reducing socioeconomic disparities) [2,12,19,26,27,29,34].

## 2. Materials and Methods

### 2.1. The Research Question

To our knowledge, no systematic reviews have been published to date on the relationship between body- or weight-related blame, guilt, and shame and mental health outcomes among children and adolescents with overweight or obesity. Therefore, the aim of this systematic review is to examine these emotions in relation to body weight and their impact on psychological functioning within the pediatric population (childhood and adolescence) affected by overweight and obesity. The research question therefore concerned the relationship between blame, guilt, and shame related to body and body weight and the psychological functioning among the pediatric population with overweight and obesity.

This systematic review employed a PICOS framework focusing on children and adolescents with overweight or obesity (Population), exploring the experiences of body- and weight-related blame, guilt, and shame (Exposure), and assessing their impact on psychological functioning (Outcomes), using original research articles (quantitative and/or qualitative studies); excluded were editorials, reviews, and commentaries (Study design).

It was assumed that the above-mentioned emotions would be associated with both intrapsychic and interpersonal functioning difficulties and would be associated with difficulties in conducting an effective therapeutic process of obesity treatment (e.g., relationships between blame, guilt and shame related to body and body weight and such aspects of intrapsychic functioning as, for example, emotional functioning, mental disorders, self-esteem, sense of efficacy, mindfulness, as well as such aspects of interpersonal functioning as, for example, communication about the body and obesity treatment between the patient and healthcare providers/child and parent, bullying and teasing history, weight stigma).

In order to verify the aforementioned aim of the study and to respond to the research question posed, the following search query was used: (blame OR guilt OR shame OR body shame OR body shaming OR weight shame) AND (mental health OR psychological health OR psychological functioning OR psychological factors) AND (children OR adolescents OR pediatric population OR pediatric sample) AND (obesity OR obese OR excessive body weight OR overweight). This systematic review was not registered in any registry. This systematic review was conducted in accordance with the Preferred Reporting Items for Systematic Reviews and Meta-Analyses (PRISMA) guidelines.

### 2.2. Finding Studies

A literature search of articles published in English was conducted via Web of Science and PubMed in June 2023 and in March 2025. Two independent researchers (KCB and MZ) screened the results. The search was conducted without restrictions on the year of publication. The inclusion criteria were as follows: (a) studies involving pediatric samples (childhood and adolescence; however, it should be noted that in some studies, data may be obtained from caregivers reporting on the child’s experiences, or a retrospective approach may be used in which participants describe or assess their childhood/adolescent experiences) [36], (b) full-text availability, and (c) original research articles. Studies were excluded if they were editorials, letters, author replies, review articles, or if the full text was unavailable. The initial search yielded 199 results. Duplicate records were identified and removed using Mendeley Reference Manager, which offers a built-in duplicate detection tool. After screening abstracts, 29 articles were selected for full-text analysis. This systematic review was not registered in any registry.

### 2.3. Quality Assessment

In order to evaluate the studies’ methodological quality, the National Institutes of Health/National Heart, Lung and Blood Institute (NIH/NHLBI) quality assessment tool for observational cohort and cross-sectional studies, NIH/NHLBI quality assessment tool for before–after (Pre-test–Post-test), and CASP Qualitative Checklist were used. All of the studies assessed with the use of NIH/NHLBI quality assessment tool for observational cohort and cross-sectional studies and NIH/NHLBI quality assessment Tool for before–after (Pre-test–Post-test) were rated as fair. The quality of 4 studies assessed with the use of CASP Qualitative Checklist was rated as high and the quality of 2 studies was rated as moderate to high.

## 3. Results

**Figure 1:**
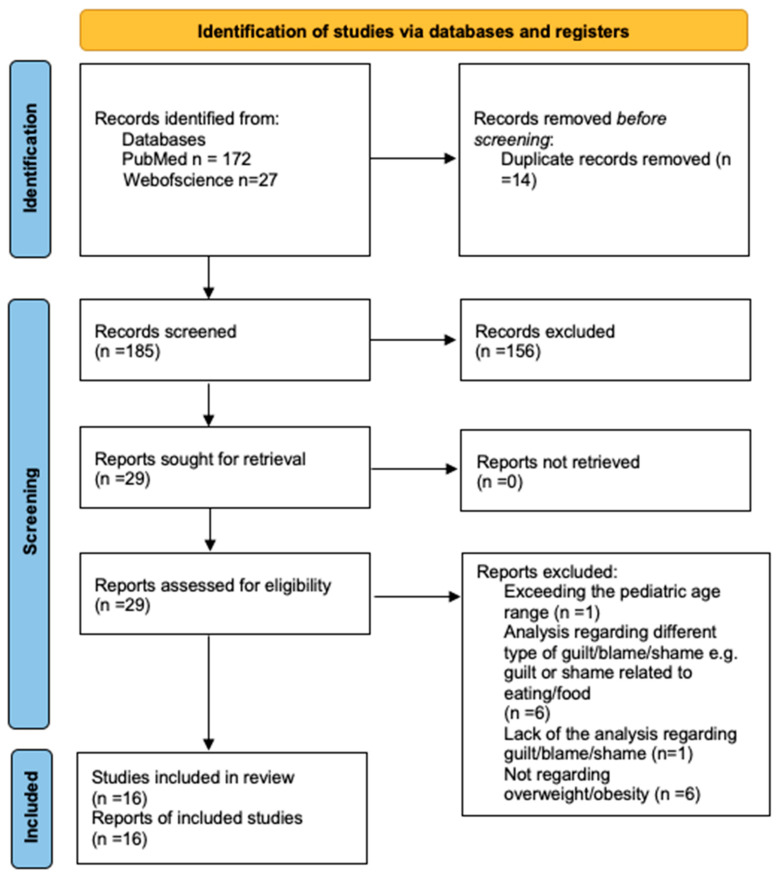
PRISMA 2020 flow diagram.

Ultimately, 16 full-text articles met the inclusion criteria and were included in the study. A flowchart outlining the study selection process is presented in Figure 1, while the characteristics and findings of the included studies are summarized in Table 1. The following information was extracted from the original articles: authors, year of publication, region, study design, participant characteristics (including sex and age), examined variables, methods, and key outcomes.

In summary, the majority of the analyzed studies employed a quantitative approach (*N* = 8), while qualitative research accounted for 37.5% (*N* = 6). The smallest category comprised mixed-methods research (*N* = 2). The oldest study included was published in 2005, with the highest number of publications occurring in 2017 and 2018 (*N* = 7). The majority of studies originated from the United States (*N* = 8), followed by Sweden and Portugal (each contributing two studies), while Canada, Italy, Norway, and China each had one publication. Most studies focused on adolescents, with only two studies examining younger children (aged 8–9 years) [19,37]. Additionally, only one study explored the childhood experiences of current adults [11], and just one explicitly presented the perspective of medical providers [13]. The vast majority of studies (*N* = 11) addressed the concept of body- or weight-related shame, while guilt and blame were examined with similar frequency. Notably, only one study analyzed all three constructs—blame, shame, and guilt—simultaneously [10]. Regarding sample characteristics, women were overrepresented in 12 studies. Additionally, most studies (*N* = 10) focused on individuals who had already undergone or were currently participating in an intervention (e.g., pediatric weight management programs, weight loss camps, bariatric surgery candidates, or individual nutrition consultations). Only four studies included parent–child dyads, whereas the remaining studies focused exclusively on children, without analyzing interactions with caregivers.

**Table 1:** Characteristics of studies regarding pediatric population with overweight and obesity.

Authors, Year and Region	Design	Participants	Sex and Age	Variables	Methods	Outcomes ^1^
**Iannaccone et al., 2016** **[38]** Italy	quantitative study	*N* = 222 *N*_O_ = 111 *N*_NW_ = 111 Students from public high schools in Southern Italy O: BMI ≥ 95th percentile for age	O: female = 43 (the rest are men) NW: female = 43 (the rest are men) 13–19 years old	* Parental bonding * Self-esteem * Shame * Perfectionism * Eating disturbance	* The Parental Bonding Instrument * The Rosenberg Self Esteem Scale * The Experience of Shame Scale * The Multidimensional Perfectionism Scale * Eating Disorder Risk Composite	O: bodily shame is significantly associated with ED risk. O: bodily shame was a significant predictor of ED risk, showing a positive relationship in the mediation model, O: self-esteem was a significant predictor of bodily shame, demonstrating a negative relationship in the mediation model
**Øen** **et al., 2018** **[10]** Norway	qualitative study	*N*_O_ = 5 Adolescents who have contact with healthcare professionals about obesity O: >30-isoBMI	80% female 12–15 years old	* Experiences in everyday life with obesity * Sense of condition * Challenges and motivation for changing behavior * Experience health-care encounters	Individual in-depth interviews (a semi-structured interview guide)	* Three key themes emerged regarding obesity: (1) a multi-faceted and challenging condition to address; (2) a source of shame and vulnerability; (3) a factor contributing to bullying and fragile social relationships. * Lack of support from parents, trusted friends, and healthcare providers—as well as experiences of bullying, shame, guilt, and self-blame—posed significant threats, reducing motivation for seeking help and making successful lifestyle changes. * The girls in our study expressed low self-confidence and self-esteem, with most interviewees reporting feelings of shame and self-blame. These emotions appeared to heighten their sense of hopelessness and acted as barriers to seeking help and implementing lifestyle changes. * A prominent theme in the interviews was the deep sense of shame young people felt about their condition and their struggle to discuss obesity-related issues with others. Despite this difficulty, they clearly expressed a need to talk about their weight concerns. They wished for others to take their struggles more seriously and were distressed by how their condition was sometimes discussed. * Support providers should avoid placing excessive demands on weight loss, as adolescents feared failure. Unrealistic expectations could intensify their feelings of shame, responsibility, and the potential loss of support.
**Orvidas ** **et al., 2020** **[19]** United States	mixed methods (qualitative and quantitative study)	T0: *N* = 9 T1: *N* = 48 T0 (intervention development): 6 undergraduate students and 3 children (8 years old) T1 (intervention testing): youth with obesity O: BMI ≥ 95th percentile	T0: NI T1: 54.17% female T0: 8–18 years old T1: 9–17 years old	* Mindsets of health * Self-efficacy * Value of health behaviors * Perceived primary control scale for children—health behaviors * Self-blame * Body dissatisfaction	* 4-item scale for mindsets of health * 10-item scale for self-efficacy * 9-item for value of health behaviors * The Perceived Control Scale for Children - adapted this measurement scale to health-related statements * Two separate prompts: “How responsible are you personally for your health? That is, how much do you feel that your health is a result of choices you make, rather than something you can’t control?”, “How responsible are you personally for your body weight? That is, how much do you feel that your body weight is a result of choices you make, rather than something you can’t control?”. * Two items from the Eating Disorder Examination Questionnaire to measure body dissatisfaction: “How dissatisfied have you felt about your weight?” and “How dissatisfied have you felt about your shape?” * Three writing activities related to growth themes	* Blame was positively correlated with perceived control and intrinsic value, while self-efficacy was positively correlated with intrinsic value. * At post-test, a growth mindset of health was positively correlated with self-efficacy, perceived control, and blame, all of which were also positively correlated with each other. * Growth mindsets related to health and health behavior cognitions (including nutrition and exercise self-efficacy and perceived control) increased significantly. However, despite efforts to reduce feelings of culpability, blame also increased from pre-test to post-test. * Contrary to expectations and previous research, participants reported significantly more blame for their current body weight at post-test compared to pre-test.
**Brewis ** **et al., 2018 ** **[39]** United States	quantitative study	*N*_baseline_ = 1443 *N* = 362 completed all four rounds of data collection College students living in first-year residence halls at a single university (Arizona State University) O: BMI ≥ 30	64.6% female at baseline 71.5% female after all four rounds of data collection first-year (freshman; no other information on the age of the participants)	* Depressive symptom levels * Body shame * Openness to friendship	* American College Health Association validated protocols— depressive symptom levels * Question about body shame from Vartanian and Shaprow’s study (2008) * Ten statements reflecting openness to friendship	* Students with overweight or obesity had higher mean levels of body shame compared to normal-weight students (Phase 1—early fall). * Students with overweight or obesity who did not experience body shame had lower mean depression scores compared to those with overweight or obesity who did experience body shame.
**Puhl and Himmelstein, 2018** **[40]** United States	quantitative study	*N* = 148 O: 34.5% OV: 37.2% Adolescents enrolled in a national weight loss camp OV: BMI = 85th to <95th percentile O: BMI ≥ 95th percentile	50% female 13–18 years old	* Perceptions of parental use of words to describe body weight * Parental comments about weight * Experienced weight stigma from family members * Internalized weight stigma	* 18 words describing body weight (+ for each word, adolescents could select whether parental use other word made them feel “sad”, “embarrassed” and/or “ashamed”, “not sure”, “fine, it doesn’t bother me” or “my parent(s) does not use this word.”) * How often does your mother [father] make comments to you about your weight? * Adolescents were asked a yes/no question to indicate whether or not their family members had teased or treated them unkindly because of their weight. * Weight Bias Internalization Scale	* Similarly, over 40% of adolescents reported feeling shame when parents referred to their body weight using terms like “fat”, “higher body weight”, “large”, “high BMI”, or “big.” * Table 3 [40] shows that words such as “large” (46.9%), “higher body weight” (46.9%), and “high BMI” (46%) were most commonly associated with feelings of “ashamed.”
**Puhl ** **et al., 2017** **[33]** United States	quantitative study	*N* = 50 Adolescents enrolled in a commercial summer weight-loss camp (Wellspring Academies) in 2016 O: BMI ≥ 95th percentile	54% female *M* = 17.28 ± 2.62 years old	* Weight-based language preferences among adolescents	* 16 words used to describe excess body weight (“If your parent(s) use any of the following words to talk about your weight, how does it make you feel?”, resp. “sad”, “embarrassed”, “ashamed”, “not sure”, or “fine, it doesn’t bother me.”)	* A large proportion of participants, particularly girls, reported feeling sadness, shame, and embarrassment when parents used certain words to describe their body weight, highlighting the importance of considering the emotional impact of weight-based terminology. * For both males and females, approximately 30% felt ashamed when parents described their weight as “obese”. * Table 2 [33] shows the emotional responses of males and females to parents using various words to describe their body weight. The five words most often associated with “ashamed” were: “ashamed” (m: 30.4%, f: 29.6%), “heavy” (m: 26.1%, f: 22.2%), “big” (m: 21.7%, f: 29.6%), “overweight” (m: 21.7%, f: 29.6%), and “weight problem” (m: 17.4%, f: 40.7%). * Our findings suggest that adolescents may react to weight-based terminology in ways that induce emotional distress. A significant percentage of adolescents, particularly females, reported feeling sad, embarrassed, and ashamed in response to parental use of certain words regarding their body weight.
**Roberts ** **et al., 2021** **[6]** United States	qualitative study	*N*_parent_ = 19 *N*_adolescent_ = 12 Adolescents with severe obesity and their parents who attend a paediatric weight management O: BMI ≥ 120th percentile of the 95th percentile of BMI for age and sex	Parent: 89.47% female 30–54 years old Adolescent: 41.67% female 12.2–16.8 years old	* Experiences of weight stigma in adolescents with severe obesity and their parents	* A secondary analysis on 31 transcripts from a larger study of 46 transcripts conducted between February 2019 and June 2020 (semi-structured interviews)	* Four common themes emerged in experiences of weight stigma: weight-based teasing and bullying, interactions with healthcare providers (HCPs), family interactions, and blame. Subthemes included perceptions of fairness and the impact on mental health. * Blame is defined as the internal attribution of weight gain to oneself or one’s adolescent, as well as experiences of being blamed by others (e.g., parents, healthcare providers, or spouses) for the adolescent’s weight. * In describing their interactions with healthcare providers (HCPs) at the pediatric weight management (PWM) clinic, most adolescents and parents reported primarily positive and supportive experiences. However, some parents and adolescents felt that certain interactions made them feel “nervous”, as if their weight was “my fault” or that their efforts to change their eating and physical activity habits were not “good enough.”. Adolescents sometimes perceived that they were “getting into trouble”, which led to feelings of anxiety and personal blame for their weight. Similarly, parents reported feeling defensive and blamed for their actions, which they believed influenced their adolescent’s weight. * Negative interactions and the consequences of stigma often led adolescents and parents to internalize blame for the child’s weight or weight gain. Additionally, they reported feeling blamed by others, including parents, healthcare providers, and spouses. * Adolescents primarily blamed themselves for their weight, often attributing it to their own actions or eating habits. A 12-year-old boy (C5) shared, “…and it is my fault [long pause and looks down] for the weight gain.” In some cases, adolescents also assigned blame to others. A 14-year-old girl (C15) expressed frustration toward her mother for purchasing unhealthy food, stating, “I’d eaten it [the cake], so my mother yelled at me. But she bought it, so she can’t really yell at me… If they wouldn’t buy ice cream, I wouldn’t eat the ice cream… That doesn’t make sense.” * Parents expressed a sense of blame for their adolescent’s weight, using phrases such as “It’s all on me”, “It’s my responsibility”, and “It’s my fault.” Many also described feeling as though they had failed their adolescent. * A mother (P2) of a 12-year-old boy, struggling with how to help her son, expressed her frustration: “Why can’t I fix this? …I feel like we failed him in some way. How did we get this far?” * Parents reported feeling blamed by both spouses and healthcare providers (HCPs), who directly attributed responsibility to them for purchasing groceries and preparing meals at home. Blame from HCPs left parents feeling discouraged and misunderstood, while blame from spouses created tension between partners. Many mothers, in particular, described feeling overwhelmed by the heavy burden of managing their own work, daily household responsibilities, and the primary responsibility for their child’s weight management. These ongoing experiences compounded feelings of blame and household stress over the years. One mother of a 16-year-old girl (P1) described the constant pressure of daily weight management: “It’s all on me. It is my responsibility.” Another mother (C14) of a 13-year-old girl—who works seven days a week and whose husband has health issues— shared her frustration: “It’s my fault, but they don’t realize how much I have to do at the house, whether it’s the laundry and the dishes, and I’m working all day.”
**Rosenberger et al., 2007** **[11]** United States	mixed methods (qualitative and quantitative study)	*N* = 174 Bariatric surgery candidates O: (NI)	75% female *M* = 42.9 ± 11.1 years old (analyzing childhood history)	* Childhood history of being negatively teased (i.e., being made fun of) about weight * Psychiatric History * Weight and eating concerns * Psychological functioning	* The Structured Clinical Interview for DSM-IV * Additional semi-structured interview * Childhood Weight, Dieting, and Teasing History: participants were asked the extent to which they were teased about weight as a child, rated using a 5-point scale ranging from ‘not at all’ to ‘extremely’ * The Eating Disorder Examination-Questionnaire * The Rosenberg Self-Esteem Scale * The Beck Depression Inventory * The Body Shape Questionnaire * The Internalized Shame Scale	* Comparison of weight history and current functioning between groups with and without a history of teasing: - Participants with a history of teasing reported significantly higher concerns related to ED (EDE-Q eating, weight, and shape concerns). However, they did not differ significantly in terms of binge eating frequency or dietary restraint. Additionally, participants who experienced childhood teasing had significantly lower self-esteem and higher scores for depression, body dissatisfaction, and shame, even after controlling for the childhood onset of obesity. - The present study also found that shame, a dimension of psychological functioning reflecting internal feelings of worthlessness and inadequacy, is significantly associated with a history of teasing. - Finally, although the two groups did not differ significantly in rates of lifetime or current psychiatric disorders, bariatric surgery candidates with a history of teasing reported significantly lower self-esteem and significantly higher levels of depression and shame. Since these findings persist even after controlling for childhood onset of obesity, it suggests that the association between teasing history and current functioning is not simply due to an earlier age of obesity onset.
**Sjöberg** **et al., 2005** **[41]** Sweden	quantitative study	*N* = 4703 O: 2.79% PreO: 13.72% Adolescent who answered the Survey of Adolescent Life in Vestmanland 2004 O and PreO: The international age- and gender-specific BMI cutoff points for children developed by the Childhood Obesity Working Group of the International Obesity Task Force14 were used to define subjects as normal weight, overweight (preobese), or obese. These cutoff points were derived from the data of a number of large international cross-sectional growth-study surveys. Cutoff points were determined through centile curves that at 18 years were drawn through the widely accepted cutoff points, 25 and 30 kg/m^2^, for adult overweight and obesity, respectively.	49.18% female 15–17 years old	* Depressive symptoms and depression * Shame and psychosocial and economic status	* A self-rating scale (Depression Self-Rating Scales) of the Diagnostic and Statistical Manual of Mental Disorders, Fourth Edition * Shame index: Have you during the latest period of 3 months experienced that someone: 1. treated you in a degrading manner? 2. made fun of you in front of others? 3. questioned your sense of honor? 4. talked about you in a degrading manner? 5. ignored you or behaved as if you did not exist? * A family-economy index: Whether the family owned a computer; had Internet access; owned a house in the country for leisure activities; owned a boat big enough to sleep in; owned a caravan; or used to go for vacations in foreign countries and go alpine skiing on vacations. * The survey also included questions regarding parental separation and parental employment.	* Obesity was also significantly associated with experiences of shame. * All significant associations between BMI grouping and depression disappeared when shaming experiences, parental employment, and parental separation were controlled for. * Adolescents who reported frequent experiences of shame had an increased risk of depression.
**Tan ** **et al., 2018** **[42]** China	quantitative study	*N*_NM_ = 1021 *N*_OV_ = 700 *N*_O_ = 321 Adolescents with normal weight, overweight status, and obese status from middle schools in urban areas located across seven geographic districts in China OV and O: The cutoff points for BMI for overweight and obesity in the Chinese BMI reference conducted by the Group of China Obesity Task Force (GCOTF)	NM: 71.50% male OV: 71.43% male O: 71.65% male NM: 11–20 OV: 11–20 O: 11–19	* Cognitive emotion regulation	* Cognitive Emotion Regulation Questionnaire	* Adolescents in the obesity group scored the highest on self-blame and rumination among the three groups. They also scored lower on acceptance, positive refocusing, and positive reappraisal compared to those in the normal weight group. * Higher scores on self-blame and rumination were associated with a higher BMI, while greater acceptance and positive refocusing were linked to a lower BMI.
**Williams ** **et al., 2008** **[32]** United States	qualitative study	*N* = 16 children with parents OV: 31% described themselves as “overweight” Ninth grade students in four high schools throughout West Virginia OV and O: NI (Healthy weight was also defined as a number on the scales that should be proportionate to one’s height. This number was defined by expert opinions, such as those conveyed by their healthcare providers or other accepted standards, such as BMI.)	Children: 44% male 14–18 years old Parents: NI	* Cultural perceptions of a healthy weight	* Separate focus group interviews were conducted concurrently with adolescent and parents or caregivers to identify the cultural perceptions of a healthy weight. Questions were developed using grounded theory to explore how a healthy weight was defined, what factors dictate body weight, the perceived severity of the obesity issue, and the social or health ramifications of the condition.	* Female participants were more concerned with their weight than males, with some expressing concerns that bordered on obsession. Both males and females described experiencing social stigma associated with overweight. * Students, particularly females, expressed various psychological consequences of overweight, including feelings of guilt and diminished self-esteem. Additionally, a reduced sense of belonging among peers and family was perceived as a consequence of obesity.
**Moreira and Canavarro, 2017** **[37]** Portugal	quantitative study	*N* = 153 children/adolescents with overweight or obesity Adolescents with overweight/obesity followed in individual nutrition consultations OV: BMI between the 85th and the 95th percentile O: BMI equal or above the 95th percentile	62.7% female 8–18 years old	* Mindfulness * Body shame * Quality of life	* The Child and Adolescent Mindfulness Measure * the body shame subscale of the Experience of Shame Scale * Portuguese child-report version of KIDSCREEN-10 index	* A strong negative correlation was found between mindfulness and body shame, as well as between quality of life and body shame in the full sample. * A negative correlation was found between mindfulness and body shame, as well as between body shame and quality of life in girls. In boys, a negative correlation was observed between mindfulness and body shame. * The moderated mediation analyses revealed that the path from body shame to quality of life was moderated only by gender. Specifically, while the interaction between body shame and gender was statistically significant, the interaction between body shame and age was not. Simple slope analyses demonstrated that an increase in body shame was associated with a decrease in quality of life only in girls.
**Morinder ** **et al., 2011** **[9]** Sweden	qualitative study	*N*_O_ = 18 Adolescents with obesity from a pediatric obesity clinic in Sweden O: Classified as obesity according to the international age- and gender-specific BMI cutoff points established by the International Obesity Task Force	66.67% female 14–16 years old	* semi-structured interviews with open-ended questions	* Semi-structured interviews with open-ended questions (the questions focused on three areas: the participant’s perceptions and understandings of obesity and weight loss, referral to the paediatric obesity clinic, participation in obesity treatment)	* Regular clinic visits require sacrifices from parents, such as arranging transportation and taking time off from work, which can reinforce feelings of guilt. Shame and guilt are also mentioned when parents are held responsible for unsuccessful treatment outcomes. * Registration and treatment result in constant focus on body weight. When weight loss is not achieved, feelings of shame, failure, and disappointment arise. * Weight gain prompts feelings of embarrassment and shame. In such cases, individuals may avoid confrontation, often leading to missed appointments. This avoidance reinforces feelings of failure and guilt. As one participant shared: “I felt ashamed… because you said you would lose weight, but instead, you gained… I felt a bit embarrassed… and you could be sort of scared, like… (IP 9).”
**Gouveia ** **et al., 2018** **[43]** Portugal	quantitative study	*N* = 572 dyads (mother or father and adolescent) Adolescent: OV and O: 43.5% Sample collected in three public school units and three pediatric public hospitals in the central region of Portugal OV and O: BMI ≥ 85th percentile according to the WHO Child Growth Standards	Adolescent: 59.09% fame 12–18 years old Mother/father: 77.8% female 30–61 years old	* Mindful Parenting * Self-compassion * Body shame * Emotional eating	* Portuguese version of IMP Scale * The Self-Compassion Scale * the body shame subscale of the Experience of Shame Scale * the Dutch Eating Behavior Questionnaire	* Adolescents with overweight/obesity undergoing nutritional treatment exhibited higher levels of body shame compared to those not undergoing treatment and those with normal weight. * Adolescents with overweight/obesity not undergoing nutritional treatment exhibited higher levels of body shame compared to adolescents with normal weight. * Mindful parenting, particularly the dimension of compassion for the child, was indirectly associated with emotional eating through adolescents’ self-compassion. This association was mediated sequentially by both self-compassion and body shame. Specifically, both adolescent boys and girls with higher levels of self-compassion had lower levels of emotional eating, and this relationship was mediated by reduced body shame. * Adolescents’ body shame was significantly and negatively correlated with mindful parenting and self-compassion, while body shame was significantly and positively correlated with emotional eating.
**Giles et al., 2023** **[16]** Canada	qualitative study	*N*_O_ = 5 5 children with spina bifida (SB) and 5 of their parents Children with SB and their parents recruited from outpatient multidisciplinary specialized SB clinics at two urban academic children’s hospitals in Canada. O: children who had previously discussed their weight with a healthcare provider at their SB clinic	60% female 10–16 years old	* semi-structured, individual interviews	* Semi-structured, individual interviews conducted in-person and via videoconference. Sample interview questions for children: 1. What do you think it means to be healthy, and what it means to be unhealthy? 2. Tell me about a time when something happened that made you feel good about your body? 3. Who talks to you about things to do with your health? What do you talk about? 4. Do you ever talk about things like food or exercise or weight when you see your doctor or nurse? How does that make you feel? 5. What is the best thing doctors or nurses can do to help keep you healthy? Sample interview questions for parents: 1. What do you think it means for your child to be healthy, and unhealthy? 2. Who talks to your child about things to do with their health? 3. What have been your experiences of speaking with healthcare providers about your child’s weight? 4. How does your child feel about discussing their health? Their weight? 5. Do you and/or your child ever talk about things like food or physical activity or weight with healthcare professionals? How do you/your child feel? 6. What are the most important things to consider when designing weight management/healthy behaviour supports/services for children with spina bifida?	Embarrassment and guilt * Two of the girls reported feeling embarrassed and guilty when discussing weight with a healthcare provider: “I feel like I’m not doing enough [physical activity and eating healthy] and that I should, like, try to do more.”
**Darling ** **et al., 2023** **[13]** USA	qualitative study	*N* = 11 Pediatric medical providers in the New England area that refer adolescents to outpatient weight management (WM) interventions. Providers were eligible to participate if they self-identified as primarily treating adolescents and reported that at least one-third of their patient population was from a low-income background (per provider report using public insurance as a proxy for income). O: NI	82% female *M* = 45.9 ± 10.3 years old	* semi-structured individual interviews	* Semi-structured individual interviews—three main topic areas: (1) experiences working with adolescents from low-income backgrounds, (2) perceptions of interactions concerning WM, and (3) barriers and facilitators to WM engagement.	* In addition to general hesitation and logistical barriers to engaging in weight management (WM), many providers also reported that adolescents and caregivers may experience guilt or shame related to the need for a referral to WM. * Providers perceive that these experiences of shame often hinder a family’s likelihood of seeking further treatment. For instance, one provider (male, MD, 54 years old) stated, “There is a lot of guilt associated with weight and weight gain… not just from the child’s perspective, but also from the parent’s perspective as well.” * Providers described significant shame associated with high weight. They often reported that adolescents experience stigma related to their weight both at home and at school, making it particularly emotional to discuss in the medical office. * Nearly all providers discussed techniques they use to reduce stigma around weight, primarily by decreasing the focus on weight and increasing the emphasis on health behaviors. * Providers also emphasized the importance of supporting adolescents’ own health priorities, rather than focusing on caregivers’ goals. For example, one provider (female, MD, 56 years old) explicitly highlighted family responses to these terms, stating, “It can still feel shaming for kids and teens when I’m telling them I’m worried about their weight– that they’re at an unhealthy weight.” * Overall, providers focused on improving communication and minimizing the negative impacts of weight-related discussions, while acknowledging the tension created by the weight-related health outcomes they observe in patients. * Despite this, providers sometimes used language to describe weight status that could be interpreted as blaming or shaming toward adolescents and caregivers. For example, one provider (female, MD, 55 years old) described caregivers, saying, “They don’t take responsibility for the changes… how do you get the parents to take responsibility?”

Note. ^1^ In many cases, in order to accurately reflect the nature of the results obtained, the results described constitute quotes of the exact description of the results by the authors of a given work; f—female, m—male, ED—eating disorders, NI—no information, NM—normal weight, O—obesity, OV—overweight, PreO—preobesity, SB—spina bifida, WM—weight management.

## 4. Discussion

Our systematic review aimed to analyze blame, guilt, and shame related to body weight and their relationship with psychological functioning among children and adolescents with overweight and obesity. Despite the critical importance of these factors in designing effective interdisciplinary prevention strategies, as well as in the diagnosis and treatment of obesity, there remains a limited number of studies examining these variables and their interconnections.

Analysis of the collected records revealed associations between body- or weight-related blame, guilt, and shame and various aspects of psychological functioning in the pediatric population. These associations were observed across the following domains: (a) interpersonal context (e.g., [6,9,10,11,13,16,32,33,40,43])—including factors such as social stigma, bullying, teasing history, social connectedness, expectations regarding weight change, sense of belonging among peers and family, weight-related language used by parents in conversations with children and adolescents, and mindful parenting; (b) intrapsychic context (e.g., [11,19,38,43])—relationship with eating and food—encompassing behaviors such as binge eating, dietary restraint, emotional eating, and the risk of developing eating disorders; (c) intrapsychic context (e.g., [10,11,19,32,43])—self-perception—including self-esteem, feelings of worthlessness and inadequacy, self-compassion, self-efficacy, perceived control, and intrinsic self-worth; (d) intrapsychic context (e.g., [11,33,39,41,42])—emotional functioning—involving emotional distress, anxiety, depression, emotion regulation strategies, and feelings of hopelessness; (e) intrapsychic context (e.g., [6,9,10,13,37])—additional psychological factors—such as mindfulness, quality of life, willingness to seek help, and motivation for both help-seeking and sustaining successful lifestyle changes.

Building on the above findings, it is important to emphasize that childhood and adolescence are critical developmental periods in which a sense of belonging within family and peer groups plays a fundamental role. The significance of social relationships for healthy development has been extensively discussed by Erikson [44] and Bowlby [45]. Moreover, this stage of life is marked by heightened susceptibility to social comparison and increased sensitivity to external judgments and environmental feedback [2,27,46]. As a result, the interplay between a child’s or adolescent’s internal factors (including the intrapsychic aspects mentioned earlier) and interpersonal influences (such as interactions with parents, peers, and healthcare providers) may contribute to psychosocial challenges that significantly impact the treatment process. Therefore, when interpreting the findings of our systematic analysis, it is essential to highlight the evidence supporting several key principles that should be considered when working with children and adolescents with obesity.

First, the findings reinforce that obesity treatment in the pediatric population should adopt a family-based approach rather than focusing solely on the child or the parent in isolation [10,40,43]. This aligns with existing research and clinical guidelines advocating for family-centered interventions [47,48,49,50]. Placing the burden of change solely on the child—while the family environment remains unchanged (e.g., family members continuing to purchase and consume foods the child is advised to limit, or inconsistent health-related rules among family members)—often reinforces feelings of shame, blame, and guilt, as well as a sense of inferiority. In contrast, involving the family in the treatment process fosters several critical benefits: (a) enhancement of parental communication skills and the adoption of parenting strategies that cultivate positive and intrinsic motivation for change in the child, rather than relying on fear, shame, or other negative emotions; (b) increased parental awareness of how their behaviors and communication impact the child’s development, particularly through behavioral modeling within the parent–child relationship, as outlined in Social Learning Theory [51]; and (c) recognition of the family as an interconnected system, wherein the functioning of the entire household—including parents, siblings, and even extended family members (e.g., grandparents)—can influence the child’s ability to engage in effective obesity treatment. This perspective also considers the psychological well-being of all family members, rather than focusing exclusively on the child with obesity.

Secondly, effective obesity treatment should be grounded in a collaborative approach that involves the patient, their family, and an interdisciplinary team comprising medical professionals such as physicians, psychologists, psychiatrists, dietitians, and physiotherapists. This multidisciplinary framework allows for a comprehensive assessment of the patient’s and family’s biopsychosocial well-being, facilitating the development of tailored interventions that address their specific needs [6,9,13,16,43]. Beyond parental awareness and skills, another critical determinant of treatment success is the communication and motivational proficiency of all members of the interdisciplinary team. This includes the ability to use Person-First Language (e.g., referring to a “patient with obesity” rather than an “obese patient”) and to engage in motivational strategies that foster patient autonomy and self-efficacy [10,33]. The importance of these competencies is underscored by initiatives such as the European Association for the Study of Obesity [52] and other professional organizations [53], as well as the growing emphasis on Motivational Interviewing as a key technique for facilitating behavioral change in obesity management [54].

Øen et al. [10] highlight that body- and weight-related blaming, shaming, and guilting can originate from both parents and healthcare providers, often stemming from explicit and implicit weight biases toward patients with obesity [6,24,55,56]. These biases, in turn, negatively impact both patient and provider engagement in the treatment process, leading to lower adherence, increased distrust, and diminished perceptions of care quality among patients [24,57,58]. Given the short- and long-term consequences of these experiences on children’s psychological well-being, it is crucial to modify existing treatment approaches to mitigate these harmful effects. A key consideration in this regard is the recognition of obesity as a chronic, relapsing, and lifelong disease [52]. Consequently, treatment efforts should prioritize sustainable behavioral changes and overall health improvements, rather than focusing exclusively on weight loss as the primary marker of success [9,10]. This perspective underscores the importance of long-term treatment strategies that emphasize health status and progress in habit modification, rather than unrealistic expectations of rapid weight reduction, which can significantly increase the risk of unhealthy behaviors such as very low-calorie dieting, fasting, vomiting, and compulsive exercise [10,13,59]. As Øen et al. emphasize, excessive pressure to achieve substantial weight loss can instill fear of failure in adolescents, exacerbating feelings of shame, self-blame, and helplessness, ultimately discouraging engagement in the treatment process [10]. This can foster detrimental self-perceptions, such as: “It’s my fault, but I don’t know how to change it, and I’m afraid change is impossible because I can’t control myself. I’m worthless.” (clinical observation from the authors’ own experience). Such beliefs contribute to avoidance behaviors, reluctance to seek help, and withdrawal from treatment efforts [60,61].

Furthermore, Gouveia et al. underscore the critical role of effective communication in obesity treatment, stating, “Weight gain prompts feelings of embarrassment and shame. In this case, one does not want to be confronted, and it is common to ‘fail’ to attend booked appointments” [43]. This notion is further supported by patient-reported experiences in Morinder et al., which illustrate how shame and fear of judgment lead adolescents to avoid medical appointments: “I felt ashamed… because you said you would lose weight but instead you gained… felt a bit embarrassed… and you could be sort of scared, like…” (IP 9) [9]. “It was summer when I’d put on… like… five kilos… then I called and cancelled on my own… I really did not want to come… did not show the appointment letter to my mother…” (IP 12) [9]. These testimonies emphasize the importance of fostering an open, nonjudgmental dialogue with patients, reinforcing that meetings with the interdisciplinary team are not assessments of success or failure, but opportunities to adapt and refine treatment strategies. Acknowledging that progress is not linear and that trial-and-error is a natural part of the process can help reduce all-or-nothing thinking and support sustained patient engagement.

Thirdly, assessing the patient’s mental state and providing psychological and psychiatric support during the treatment process should be fundamental components of therapeutic interventions for obesity. This is particularly important because shame, guilt, and self-blame related to body image and/or weight are associated with impaired self-esteem and self-efficacy (e.g., shame, blame, and guilt as a “resource thief”), the development and progression of mental disorders (e.g., depressive symptoms, eating disorders, and other disordered eating behaviors such as secretive or emotional eating), as well as a lower quality of life and the use of maladaptive emotion regulation strategies [10,19,32,37,38,39,41,42]. Moreover, the negative consequences associated with these factors may contribute to the persistence of obesity and other long-term physical and mental health complications [11]. In conclusion, it is important to emphasize that, in some cases, reducing resistance to treatment and/or alleviating symptoms of mental disorders (e.g., depression, anxiety disorders) is necessary to facilitate collaboration with other healthcare team members. Without addressing these psychological factors, effective cooperation may be significantly hindered, highlighting the critical role of collaboration between patients, parents, psychologists/psychotherapists, and psychiatrists.

Understanding the dynamics of body- and/or weight-related blame, guilt, and shame among children and adolescents with overweight and obesity is essential for developing effective support systems that enhance their well-being and psychological resilience in both the prevention and treatment of obesity. In summary, the majority of included studies were conducted using quantitative designs (N = 8), followed by qualitative (N = 6) and mixed-methods (N = 2) approaches. The oldest study was published in 2005, with a peak in 2017–2018, and originated predominantly from the United States. Adolescents were the primary population studied, with limited research on younger children. Notably, few studies examined the parent–child dyads or healthcare providers, and only one study explored all three constructs—blame, guilt, and shame—simultaneously. Female participants were overrepresented in most studies, and the majority of samples included individuals already engaged in weight-related interventions. Analysis of the collected records revealed associations between body- or weight-related blame, guilt, and shame and various aspects of psychological functioning in the pediatric population (e.g., interpersonal context: social stigma, bullying, teasing history, social connectedness, expectations regarding weight change, sense of belonging among peers and family, weight-related language used by parents in conversations with children and adolescents, and mindful parenting; intrapsychic context: relationship with eating and food—encompassing behaviors such as binge eating, dietary restraint, emotional eating, and the risk of developing eating disorders; intrapsychic context: self-perception—including self-esteem, feelings of worthlessness and inadequacy, self-compassion, self-efficacy, perceived control, and intrinsic self-worth; intrapsychic context: emotional functioning—involving emotional distress, anxiety, depression, emotion regulation strategies, and feelings of hopelessness; and intrapsychic context: additional psychological factors—such as mindfulness, quality of life, willingness to seek help, and motivation for both help-seeking and sustaining successful lifestyle changes).

Interventions aimed at pediatric obesity prevention and treatment should actively address and reduce experiences of blame, guilt, and shame to promote psychological well-being and long-term treatment success. The findings of this study highlight the need for healthcare providers, educators, and caregivers to receive training on stigma reduction. Clinical protocols should incorporate psychological screening for shame- and guilt-related distress, while schools and family-based programs should focus on promoting body acceptance.

However, our study is not without limitations. Firstly, a significant proportion of the reviewed studies originate from the United States and are based on experiences shaped by the cultural, social, and economic contexts specific to that region (which may involve the possibility of significant cultural differences). Secondly, the sample sizes and sex distribution vary considerably across studies, with most research focusing primarily on adolescence. Thirdly, the analyzed studies differ in terms of their sample composition, including both non-clinical populations (e.g., students from public high schools) and clinical populations (e.g., patients from pediatric obesity clinics). Fourthly, some studies lack even basic descriptive data about the analyzed participants (e.g., parental characteristics—Williams et al. [32]). Fifthly, the research also lacks a detailed analysis of the social support (including structural support, functional support, key aspects of support including emotional support, informational support, and practical support), as well as an analysis of different types of relationships of adolescents. Sixthly, there is a lack of analyses of long-term treatment effects achieved by patients with differences in their perceived levels of body- and weight-related shame, guilt, and blame. These limitations may significantly affect the generalizability of the findings. Therefore, further research is needed to explore the relationships between body- and weight-related blame, guilt, and shame and psychological functioning in pediatric populations with overweight and obesity, including in other regions of the world (such as Poland). Future studies—particularly longitudinal or experimental research—should address these limitations and focus more on the dynamics of child–parent–healthcare provider interactions, as well as the long-term consequences of body- and weight-related blame, guilt, and shame. Moreover, further research, including updated and expanded systematic reviews, is warranted in order to synthesize future studies in this field.

Additionally, given that guilt and blame have been studied less extensively than shame, further research should examine these aspects in greater depth. An interesting direction for future studies would be to analyze the relationship between healthcare providers’ tendencies to engage in body- and weight-related shaming or blaming and their communication skills and mental well-being (e.g., levels of professional burnout, emotion regulation skills, and personality traits). Further research is also needed on parent–child dynamics, particularly in the context of parenting skills and attitudes that support the child during obesity treatment. Additionally, future studies should consider the influence of social media on the development and reinforcement of body- and weight-related shame, guilt, and blame (also in relation to the usage/abuse of social networks and their conditioning power in the analyzed area).

## Data Availability

The original contributions presented in this study are included in the article. Further inquiries can be directed to the corresponding authors.

## Abbreviations

The following abbreviations are used in this manuscript:

BMI	Body mass index
ED	Eating disorders
IP	Interviewed person
N	Number
NI	No information
NM	Normal weight
O	Obesity
OV	Overweight
PreO	Preobesity
SB	Spina bifida
WM	Weight management
